# The Sp1-mediaded allelic regulation of *MMP13* expression by an ESCC susceptibility SNP rs2252070

**DOI:** 10.1038/srep27013

**Published:** 2016-06-01

**Authors:** Meng Shi, Jianhong Xia, Huaixin Xing, Wenjun Yang, Xiangyu Xiong, Wenting Pan, Sichong Han, Jinhua Shang, Changchun Zhou, Liqing Zhou, Ming Yang

**Affiliations:** 1 Shandong Key Laboratory of Radiation Oncology, Cancer Research Center, Shandong Cancer Hospital affiliated to Shandong University, Shandong Academy of Medical Sciences, Jinan, Shandong Province, China; 2 Beijing Laboratory of Biomedical Materials, College of Life Science and Technology, Beijing University of Chemical Technology, Beijing, China; 3 Department of Radiation Oncology, Huaian No. 2 Hospital, Huaian, Jiangsu Province, China; 4 Department of Anesthesiology, Shandong Cancer Hospital and Institute, Jinan, Shandong Province, China; 5 Key Laboratory of Fertility Preservation and Maintenance (Ministry of Education), Ningxia Medical University, Yinchuan, Ningxia, China; 6 Clinical Laboratory, Shandong Cancer Hospital affiliated to Shandong University, Shandong Academy of Medical Sciences, Jinan, Shandong Province, China

## Abstract

Metallopeptidase 13 (*MMP13*), a well-known and highly regulated zinc-dependent MMP collagenase, plays a crucial part in development and progression of esophageal squamous cell carcinoma (ESCC). Therefore, we examined associations between ESCC susceptibility and four haplotype-tagging single nucleotide polymorphisms (htSNPs) using a two stage case-control strategy. Odds ratios (OR) and 95% confidence intervals (95% CI) were computed by logistic regression model. After analyzing 1588 ESCC patients and frequency-matched 1600 unaffected controls, we found that *MMP13* rs2252070 G > A genetic polymorphism is significantly associated with ESCC risk in Chinese Han populations (GA: OR = 0.63, 95% CI = 0.54–0.74, *P* = 1.7 × 10^−6^, AA: OR = 0.73, 95% CI = 0.66–0.81, *P* = 1.8 × 10^−6^). Interestingly, the rs2252070 G-to-A change was shown to diminish a Sp1-binding site in ESCC cells. Reporter gene assays indicated that the rs2252070 A allele locating in a potential *MMP13* promoter has low promoter activities. After measuring *MMP13* gene expression in sixty-six pairs of esophageal cancer and normal tissues, we observed that the rs2252070 A protective allele carriers showed decreased oncogene *MMP13* expression. Results of these analyses underline the support of the notion that *MMP13* might function as a key oncogene in esophageal carcinogenesis.

Esophageal squamous cell carcinoma (ESCC) is one of the most common malignant tumors in the world, showing a relatively high morbidity in China^1^. Overall, ESCC patients have poor prognosis unless exhaustive treatments, including radical surgery, chemotherapy, and radiotherapy, are given. Accumulated evidences indicate that cigarette smoking, heavy ethanol consumption, micronutrient deficiency as well as dietary carcinogen exposure are main environmental risk factors of ESCC^2^,^3^. Recent progress on genome-wide association studies (GWAS) and candidate gene studies indicate that genetic makeup also contributes to ESCC etiology^4^,^5^,^6^,^7^,^8^,^9^,^10^.

As a family of zinc-ion dependent endopeptidases, matrix metalloproteinases (*MMPs*) comprise more than 21 subtypes^11^,^12^. MMPs include collagenases and gelatinases, which mediate degradation of basement membranes and the extracellular matrix^11^,^12^. Matrix metalloproteinases 13 (*MMP13*), encoded by the *MMP13* gene, is a well-known and highly regulated zinc-dependent MMP collagenase^11^. It has been reported that *MMP13* is significantly overexpressed in ESCC tissues compared with normal esophageal epithelium^12^. Deregulated *MMP13* expression might impact prognosis of ESCC patients through tumor invasion, vascular permeation, and lymph node metastasis^13^,^14^,^15^. *MMP13*, in combination with MMP7 and MMP9, are involved in early stage development of ESCC, and their co-expression predicts poor outcome for relatively early stage ESCC cases^14^. Moreover, MUC1 induces cancer cell metastasis by upregulating *MMP13* in ESCC^15^. Due to the crucial role of *MMP13* in ESCC development, it is essential to figure out the molecular mechanisms in fine-regulation of *MMP13* expression during tumorigenesis.

In the *MMP13* gene locus, multiple functional single nucleotide polymorphisms (SNPs) have been identified. One of the most studied SNPs is the *MMP13* rs2252070 polymorphism. Yoon *et al.* firstly evaluated its biological function via *in vitro* reporter gene assays and electrophoretic mobility-shift assays (EMSA) and showed that this polymorphism was a functional variant^16^. In HepG2 cells, the *MMP13* promoter with the rs2252070 A allele had approximately twice as much transcriptional activity as that with the G allele in the same position (*P* = 0.0037)^16^. EMSA using nuclear extracts prepared HepG2 cells demonstrated that *MMP13* probes containing the A allele differed in their binding to nuclear factors from the probes containing the G allele^16^. The associations between this functional SNP and multiple malignancies were repeatedly investigated in different ethnic populations^17^,^18^,^19^,^20^. However, the details on how this genetic variant impacts *MMP13* expression is still largely unknown. Considering the importance of *MMP13* in cancer development, we selected 4 haplotype-tagging SNPs (htSNP) across the whole *MMP13* locus and conducted three large independent hospital-based case-control studies to investigate the association between *MMP13* genotypes and ESCC risk. In addition, to the best of our knowledge, we firstly examined the fine-regulation of *MMP13* expression by rs2252070-mediaed allelic binding of Sp1 and its involvement in ESCC. To validate the biological function of *MMP13* rs2252070 genetic variant *in vivo*, we detected the association between its genotypes and *MMP13* mRNA expression levels in normal and cancerous esophagus tissues.

## Materials and Methods

### Study subjects

This study consisted of two case-control sets: (a) Jiangsu set: 588 ESCC cases from Huaian No. 2 Hospital (Huaian, Jiangsu Province, China) and sex- and age-matched 600 controls. (b) Shandong set: 1000 cases with ESCC from Shandong Cancer Hospital affiliated to Shandong University, Shandong Academy of Medical Sciences (Jinan, Shandong Province, China) and sex- and age-matched (±5 years) 1000 healthy controls. Sixty-six pairs of ESCC specimens and esophagus normal tissues adjacent to the tumors were obtained from surgically removed specimens of patients in Bethune International Peace Hospital and Huaian No. 2 Hospital. All individuals were ethnic Han Chinese. At recruitment, the informed consent was obtained from each subject. The detailed information on subject recruitments can be found in Supplementary Table 1 and our previous studies^21^. This study was approved by the Institutional Review Boards of Huaian No. 2 Hospital and Shandong Cancer Hospital affiliated to Shandong University, Shandong Academy of Medical Sciences. At recruitment, the written informed consent was obtained from each subject. The methods were carried out in accordance with the approved guidelines.

### SNP selection and genotyping

There are multiple SNPs in *MMP13* covering a ~13 kb region of chromosome 11q22.3. As a result, we utilized an htSNP approach to examine the *MMP13* polymorphisms globally^22^. Genotyped HapMap SNPs among Han Chinese population (HCB data, HapMap Rel 27, NCBI B36) with a minor allele frequency >5% were included in the selection. The htSNPs were chosen in a ~17 kb region (~13 kb *MMP13* locus and 2 kb up-stream as well as 2 kb down-stream regions of *MMP13*). Four htSNPs were finally selected with Haploview 4.2 software on a block-by-block basis, using a method described previously with the sample size inflation factor, Rh^2^, of ≥0.8 (Supplementary Table 2). *MMP13* htSNPs (rs11225490, rs2252070, rs17099788 and rs3758854) were analyzed by the MassArray system (Sequenom Inc., San Diego, California, USA). A 5% blind, random sample of study subjects was genotyped in duplicates and the reproducibility was 99%.

### EMSA

Synthetic double-stranded and 3′ biotin-labeled oligonucleotides corresponding to the Sp1 consensus binding sequence, *MMP13* rs2252070G or rs2252070A sequences (Supplementary Table 3) and pure recombinant Sp1 protein (E639A, Promega) were incubated at 25 °C for 20 min using the Light Shift Chemiluminescent EMSA Kit (Pierce, Rockford, IL). The reaction mixture was separated on 6% PAGE, and the products were detected by Stabilized Streptavidin-Horseradish Peroxidase Conjugate (Pierce). Unlabeled probes at 100-fold molar excess were added to the reaction mixture before the addition of biotin-labeled probes in competition assays.

### *MMP13* reporter gene constructs

Specific primer pairs (Supplementary Table 4) with *Xho*I and *Kpn*I restriction sites were used to amplify multiple deletion fragments spanning 5′-region of *MMP13* (from −186 bp to −41 bp, relative to the transcription start site) from human genomic DNA carrying *MMP13* rs2252070GG or rs2252070AA genotype. The PCR products were then digested with *Xho*I and *Kpn*I (New England Biolabs) and ligated into an appropriately digested pGL3-Basic vector (Promega) containing the firefly luciferase gene as a reporter. The resultant plasmid, designated pMMP-G or pMMP-A. Complete DNA sequencing confirmed the orientation and integrity of these two reporter constructs.

### Dual luciferase reporter assays

KYSE30 and KYSE150 ESCC cells were transfected with both reporter constructs (pGL3-Basic, pMMP-G or pMMP-A) and pRL-SV40 (Luciferase Assay System; Promega). Dual luciferase activities were examined at 48 h after transfection as previously described^23^,^24^. For each reporter construct, three independent transfections were done, and each was performed in triplicate.

### Real-time Analysis of *MMP13* mRNA

Total RNA samples from esophageal tissue specimens were extracted using TRIzol Reagent (Invitrogen) and converted to cDNA using the ReverTra Ace qPCR RT Kit (TOYOBO). *MMP13* mRNA expression was detected using the SYBR-Green real-time quantity PCR method as described previously^23^,^24^. Relative gene expression lelvels for *MMP13* and *β-actin* as an internal reference gene was carried out using the ABI 7500 real-time PCR system in triplicates. The primers used for *MMP13* were 5′-GCAAGACTCTCCTGTTCTCAGGAAA-3′ and 5′-CGGTTACTCCAGATGCTGTATTCAA-3′; and for *β-actin* were 5′-GGCGGCACCACCATGTACCCT-3′ and 5′-AGGGGCCGGACTCGTCATACT-3′.

### Western blotting

KYSE30 and KYSE150 cells were transfected with 20 nM nc RNA or Sp1 siRNAs (siSp1-1 or siSp1-2). Cells were harvested at 48 h after transfection and cell lysates were immunoblotted as previously reported^24^,^25^. Antibodies against *MMP13* (abcam, ab75606), Sp1 (Millipore, 07-645), or GAPDH (Santa Cruz, 6C5) were used.

### Statistical Analyses

Pearson’s χ^2^ test was used to examine the differences in demographic variables, smoking status, drinking status, and genotype distributions of *MMP13* SNPs between ESCC cases and healthy controls. Unconditional logistic regression model was utilized to estimate associations between *MMP13* genotypes and ESCC risk by odds ratio (OR) and their 95% confidence intervals (CIs). All ORs were adjusted for age, sex, drinking and smoking status, where it was appropriate. A *P* value of less than 0.05 was used as the criterion of statistical significance, and all statistical tests were two-sided. All analyses were performed with SPSS software package (Version 16.0, SPSS Inc., Chicago, IL).

## Results

### *MMP13* rs2252070 is associated with ESCC risk in Chinese Han populations

No Significant differences were found between cases and controls for both case-control sets in terms of median age and sex distribution (both *P* > 0.05). This indicated that the frequency matching of age and sex was adequate (Supplementary Table 1). The genotype frequencies of *MMP13* candidate SNPs (rs11225490 T > C, rs2252070 G > A, rs17099788 A > G and rs3758854 G > A) are summarized in Table 1. All observed genotype frequencies in either controls or cases conform to Hardy-Weinberg equilibrium. Distributions of the all genotypes were then compared among patients and controls. Frequencies of rs2252070 genotypes among patients differed significantly from those among controls (all *P* < 0.05). Logistic regression analyses revealed that rs2252070 SNP was significantly associated with ESCC risk (allelic OR = 0.70, 95% CI = 0.60–0.83, *P* = 2.1 × 10^−5^) (Table 1). However, no statistically significant differences of other htSNPs were observed between cases and controls (all *P* > 0.05) (Table 1). As a result, no additional analyses on these SNPs were performed in the next studies.

Associations between genotypes of *MMP13* rs2252070 G > A SNP and ESCC risk were calculated using unconditional logistic regression analyses (Table 2). The *MMP13* rs2252070 A allele was found to be a protective allele. Individuals with the rs2252070 GA genotype had an OR of 0.65 (95% CI = 0.49–0.88, *P* = 0.004) for developing ESCC in Jiangsu Set, compared with individual having the rs2252070 GG genotype. Similarly, the rs2252070 AA genotypes had a significantly decreased risk for ESCC compared with the rs2252070 GG genotype (OR = 0.79, 95% CI = 0.65–0.95, *P* = 0.011). In Shandong Set, carriers of the rs2252070 GA or AA genotypes were significantly associated with decreased ESCC risk (OR = 0.65, 95% CI = 0.53–0.81, *P* = 8.8 × 10^−5^, or OR = 0.75, 95% CI = 0.65–0.86, *P* = 4.9 × 10^−5^) (Table 3). In the pooled analyses, we observed similar results (For GA genotype: OR = 0.63, 95% CI = 0.54–0.74, *P* = 1.7 × 10^−6^, For AA genotype: OR = 0.73, 95% CI = 0.66–0.81, *P* = 1.8 × 10^−6^) (Table 2). All ORs were calculated with adjustments of sex, age, smoking and alcohol drinking status. The risk of ESCC associated with the rs2252070 SNP was further investigated by stratifying for age, sex, smoking and alcohol drinking status using the combined data of two case-control sets (Table 3). The variant genotypes of *MMP13* rs2252070 (GA or AA) were consistently associated with a significantly decreased risk of ESCC in all subgroups.

### *MMP13* rs2252070 SNP mediated allele-specific Sp1 binding in ESCC cells

**Figure 1:**
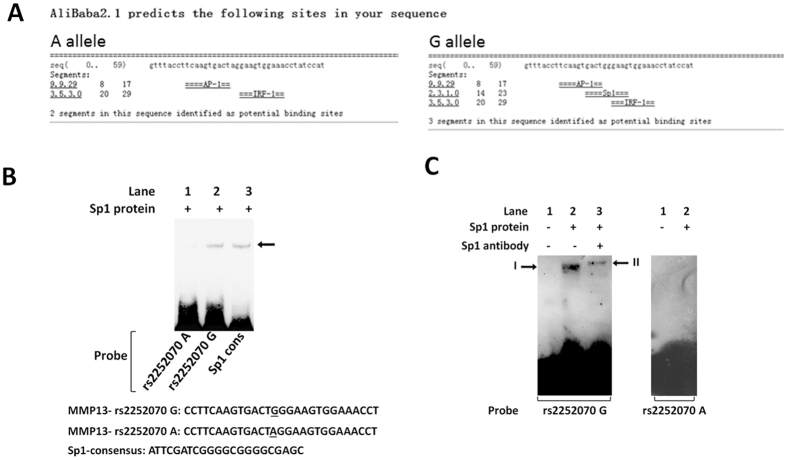
Abolishment of a Sp1 binding site in the *MMP13* promoter by the rs2252070 G > A genetic variant. (**A**) Alibaba 2.1 prediction. (**B**) Electrophoretic mobility-shift assay (EMSA) with biotin-labeled rs2252070G or rs2252070A probes and KYSE30 nuclear extracts. Lanes 1 and 6, probe only; lanes 3 and 8, probe and nuclear extracts; lanes 2 and 7, probe and nuclear extracts plus 100× unlabeled rs2252070G; lanes 4 and 9, probe and nuclear extracts plus 100× unlabeled rs2252070A; lanes 5 and 10, probe and nuclear extracts plus 100× unlabeled Sp1 consensus probes. (**C**) Super-EMSA with biotin-labeled rs2252070G or rs2252070A probes, pure recombinant Sp1 protein or Sp1 antibody. Lanes 1, probe only; lanes 2, probe and recombinant Sp1 protein; lanes 3, probe, recombinant Sp1 protein and Sp1 antibody.

Since rs2252070 SNP is located a potential Sp1 binding sequence of the *MMP13* 5′-region (Fig. 1A), we then conducted EMSA to distinguish the differences in binding capacity between the rs2252070G and A alleles to Sp1 (Fig. 1B). As shown in Fig. 1B, we found that Sp1 protein bound only to the biotin-labeled oligonucleotide probe with the G allele but not the A allele probe. We also did competition assay using pure recombinant Sp1 protein and Sp1 antibody (Fig. 1C). Interestingly, there is a super-shift band after adding Sp1 antibody, which conforming that the binding protein with *MMP13* G probe is Sp1.

### Allelic regulation of rs2252070 on *MMP13* promoter activities

**Figure 2:**
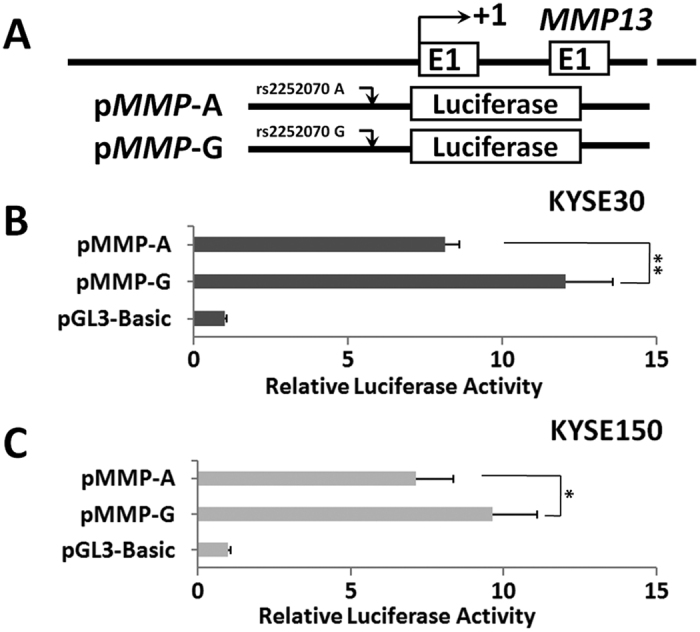
Transient luciferase reporter gene expression assays with constructs containing different rs2252070 alleles of *MMP13* 5′-region (**A**) in KYSE30 cells (**B**) or KYSE150 cells (**C**). To standardize transfection efficiency, we cotransfected pRL-SV40 with these reporter gene constructs. Fold-changes were calculated by defining the luciferase activity of cells co-transfected with pGL3-basic as 1. All experiments were performed in triplicates in three independent transfection experiments and each value represents mean ± SD. Compared with pGL3-Basic transfected cells, *P < 0.05; **P < 0.01.

Because the rs2252070 SNP is located in the Sp1 binding sit of *MMP13* promoter, we speculated that this polymorphism will influence gene expression of *MMP13*. Therefore, we examined the promoter activity of this region by two luciferase reporter constructs (Fig. 2A) with different rs2252070 allele in ESCC KYSE30 and KYSE150 cells. Interestingly, the *MMP13* rs2252070G allelic reporter construct (pMMP-G) showed significantly higher luciferase activities compared to the rs2252070A allelic reporter construct (pMMP-A) in KYSE30 cells (*P* < 0.01) (Fig. 2B). Moreover, KYSE150 cells transfected with pMMP-G showed significantly higher luciferase activities compared to cells expressing pMMP-A (*P* < 0.05) (Fig. 2C). This indicates that Sp1 could bind the rs2252070G allelic *MMP13* promoter and prompt increased *MMP13* expression.

### Impacts of rs2252070 on *MMP13* expression *in vivo*

**Figure 3:**
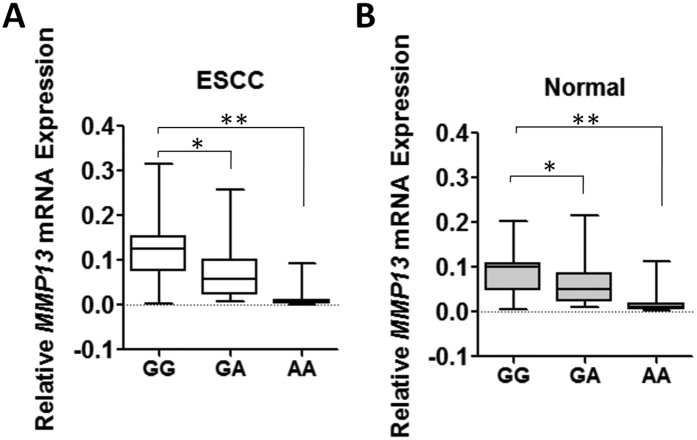
*MMP13* mRNA expression (mean ± SD) in normal and cancerous esophageal tissues grouped by *MMP13* rs2252070 genotypes. The expression of individual *MMP13* mRNA was calculated relative to expression of *β-actin* using the 2^−dCt^ method. *P < 0.05; **P < 0.01.

Considering rs2252070 G-to-A change could impact *MMP13* promoter activity in cancer cells, we investigated whether there is an allele-specific effect of rs2252070 SNP on *MMP13* expression in esophagus tissue specimens (Fig. 3). We found that there were significantly lower *MMP13* mRNA levels (mean ± SD) among carriers of the rs2252070 AA genotype compared to carriers of the GG genotype in ESCC tissues (0.014 ± 0.026 [*n* = 12] vs. 0.119 ± 0.074 [*n* = 24], *P* < 0.01) or normal esophagus tissues (0.024 ± 0.034 [*n* = 12] vs. 0.088 ± 0.050 [*n* = 24], *P* < 0.01). Similar results have also been observed for GA genotype in ESCC tissues (0.072 ± 0.058 [*n* = 30] vs. 0.119 ± 0.074 [*n* = 24], *P* < 0.05) or normal esophagus tissues (0.060 ± 0.043 [*n* = 30] vs. 0.088 ± 0.050 [*n* = 24], *P* < 0.05).

**Figure 4:**
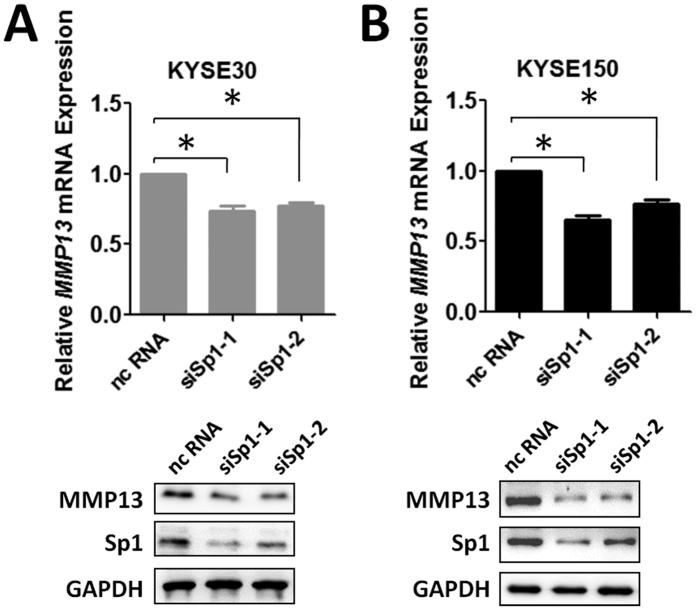
Silencing Sp1 expression repressed *MMP13* expression in KYSE30 and KYSE150 cells. The expression of individual *MMP13* mRNA was calculated relative to expression of *β-actin*. *P < 0.05. Protein expression of *MMP13*, Sp1 and GAPDH was detected using Western Blotting.

To further verify if Sp1 indeed promotes *MMP13* expression, we knocked-down endogenous Sp1 with siRNAs and examine the expression of *MMP13* in two ESCC cell lines. Decreased *MMP13* expression was observed after Sp1 silencing (Fig. 4), suggesting that Sp1 enhance endogenous *MMP13* expression in ESCC cells.

## Discussion

In the current study, we systematically examined the impacts of SNPs in the *MMP13* locus on ESCC susceptibility via a case-control design as well as gene expression of *MMP13 in vitro* and *in vivo*. After genotyping 4 htSNPs at the discovery stage, we identified one ESCC susceptibility genetic polymorphism (rs2252070) which were validated in another case-control sets. Reporter gene assays indicated that the ESCC susceptibility SNP rs2252070 a potential *MMP13* promoter has a genotype-specific effect on *MMP13* expression. Our observations support the hypothesis that genetic polymorphisms in oncogene regulatory elements might impact genetic susceptibility of ESCC.

ESCC frequently shows extensive local invasion or regional lymph node metastasis at diagnosis and, thus, is one of the most common aggressive diseases with poor outcome. Tumor invasion and metastasis require the actions of MMPs for degradation of extracellular matrix. *MMP13*, a well-known zinc-dependent MMP collagenase, has been identified as a essential MMP in facilitating ESCC development. Elevated *MMP13* expression was not only observed in ESCC tissues but also associated with tumor invasion, vascular permeation, and lymph node metastasis^12^,^13^,^14^,^15^. Therefore, it is crucial to examine fine-regulation of *MMP13* expression during esophageal carcinogenesis. Accumulated evidences demonstrated that the *MMP13* rs2252070 polymorphism is a regulatory polymorphism in cells, but the detailed mechanisms are far from clear. Here, for the first time, we found that the rs2252070 G allele but not A allele could bind Sp1 and promote *MMP13* expression in ESCC. This is consistent with our molecular epidemiology studies showing that *MMP13* rs2252070 A allele is a protecting allele of ESCC in Chinese. That is, subjects carrying the *MMP13* rs2252070 A allele without Sp1 binding have less oncogene *MMP13* expression; therefore, these carriers show decreased risk to develop ESCC.

In all, we demonstrated that functional *MMP13* rs2252070 SNP was associated with a significantly decreased risk of ESCC in Chinese Han populations. Functional analysis showed that the rs2252070A allele contributes to significantly decreased expression of *MMP13 in vitro* and *in vivo* in the target tissues, which is most likely due to a diminished Sp1 regulation. These findings constitute strong evidence in support of the notion that *MMP13* might function as a key oncogene in esophageal carcinogenesis.

## Additional Information

**How to cite this article**: Shi, M. *et al.* The Sp1-mediaded allelic regulation of *MMP13* expression by an ESCC susceptibility SNP rs2252070. *Sci. Rep.* **6**, 27013; doi: 10.1038/srep27013 (2016).

## Supplementary Material

Supplementary Information

## Figures and Tables

**Table 1:** Associations between candidate SNPs in the *MMP13* and ESCC risk in Jiangsu case-control set (Discovery set).

No	Identity	Case	Common genotype	Heterozygous genotype	Rare genotype	Allelic OR^2^ (95% CI)	*P*
1	rs11225490 T > C	ESCC	0.580	0.359	0.061	0.89 (0.74–1.08)	0.251
		Control	0.608	0.342	0.050		
2	rs2252070 G > A	ESCC	0.369	0.454	0.177	0.70 (0.60–0.83)	2.1 × 10^−5^
		Control	0.266	0.513	0.225		
3	rs17099788 A > G	ESCC	0.760	0.235	0.005	1.11 (0.87–1.41)	0.393
		Control	0.750	0.232	0.018		
4	rs3758854 G > A	ESCC	0.835	0.160	0.005	1.09 (0.82–1.44)	0.569
		Control	0.827	0.163	0.010		

Note: SNP, single nucleotide polymorphism; *MMP13*, matrix metallopeptidase 13; ESCC, esophageal squamous cell carcinoma; OR, odds ratio; CI, confidence interval.

^1^Data were calculated by logistic regression.

**Table 2:** Genotype frequencies of *MMP13* rs2252070 genetic variant among patients and controls and their association with ESCC risk.

Studies	*MMP13* rs2252070 G > A				
	Genotypes	Cases No. (%)	Controls No. (%)	OR^1^ (95% CI)	*P*^1^
		*n* = 588	*n* = 600		
Jiangsu set	GG	217 (36.9)	157 (26.6)	1.00	
	GA	267 (45.4)	308 (51.3)	0.65 (0.49–0.88)	0.004
	AA	104 (17.7)	135 (22.5)	0.79 (0.65–0.95)	0.011
		*n* = 1000	*n* = 1000		
Shandong set	GG	382 (38.2)	274 (27.4)	1.00	
	GA	463 (46.3)	513 (51.3)	0.65 (0.53–0.81)	8.8 × 10^−5^
	AA	155 (15.5)	213 (21.3)	0.75 (0.65–0.86)	4.9 × 10^−5^
		*n* = 1588	*n* = 1600		
Pooled	GG	599 (37.7)	431 (26.9)	1.00	
	GA	730 (46.0)	821 (51.3)	0.63 (0.54–0.74)	1.7 × 10^−6^
	AA	259 (16.3)	348 (21.8)	0.73 (0.66–0.81)	1.8 × 10^−6^

Note: ESCC, esophageal squamous cell carcinoma; *MMP13*, matrix metallopeptidase 13; OR, odds ratio; CI, confidence interval.

^1^Data were calculated by logistic regression with adjustment for age, sex, smoking and drinking status.

**Table 3:** Risk of ESCC associated with *MMP13* rs2252070 G > A genotypes by age, sex, smoking status and drinking status.

Variable	*MMP13* rs2252070 G > A				*MMP13* rs2252070 G > A			
	GG^1^	GA^1^	OR^2^ (95% CI)	*P*	GG^1^	AA^1^	OR^2^ (95% CI)	*P*
Sex								
Male	436/337	546/601	0.71 (0.59–0.86)	3.1 × 10^−4^	436/337	192/266	0.76 (0.67–0.85)	4.3 × 10^−5^
Female	163/94	184/220	0.47 (0.33–0.67)	3.2 × 10^−5^	163/94	67/82	0.69 (0.56–0.87)	0.001
Age (years)								
≤57	321/226	331/429	0.56 (0.44–0.70)	3.8 × 10^−5^	321/226	135/161	0.78 (0.67–0.90)	0.001
>57	278/205	399/392	0.77 (0.61–0.97)	0.026	278/205	124/187	0.71 (0.61–0.82)	6.7 × 10^−5^
Smoking status								
No	265/182	359/320	0.76 (0.59–0.96)	0.024	265/182	131/143	0.81 (0.69–0.94)	0.007
Yes	334/249	371/501	0.55 (0.45–0.69)	5.9 × 10^−6^	334/249	128/205	0.69 (0.60–0.79)	7.8 × 10^−6^
Drinking status								
No	309/206	413/420	0.65 (0.52–0.81)	1.8 × 10^−4^	309/206	131/179	0.70 (0.60–0.81)	9.0 × 10^−6^
Yes	290/225	317/401	0.62 (0.49–0.77)	3.2 × 10^−5^	290/225	128/169	0.77 (0.67–0.89)	3.7 × 10^−4^

Abbreviations: ESCC, esophageal squamous cell carcinoma; *MMP13*, matrix metallopeptidase 13; OR, odds ratio; CI, confidence interval.

^1^Number of case patients with genotype/number of control subjects with genotype.

^2^Data were calculated by logistic regression, adjusted for sex, age, smoking, and drinking status, where it was appropriate.
