# Supplementary material for: The Sp1-mediaded allelic regulation of MMP13 expression by an ESCC susceptibility SNP rs2252070
Source: Sci Rep. 2016 Jun 1;6:27013. doi: 10.1038/srep27013 (PMC4887914; doi:10.1038/srep27013)
Supplement: Supplementary Information [file srep27013-s1.doc]

**The Sp1-mediaded allelic regulation of *MMP13* expression by an ESCC susceptibility SNP rs2252070**

Meng Shi^1,2^,**^†^**, Jianhong Xia^3^,**^†^**, Huaixin Xing^4^,**^†^**, Wenjun Yang^5^; Xiangyu Xiong^2^, Wenting Pan^2^, Sichong Han^2^, Jinhua Shang^2^, Changchun Zhou^6^, Liqing Zhou^3^,*, Ming Yang^1^,*

**Authors’ affiliations:** ^1^Shandong Key Laboratory of Radiation Oncology, Cancer Research Center, Shandong Cancer Hospital and Institute, Jinan, Shandong Province, China; ^2^Beijing Laboratory of Biomedical Materials, College of Life Science and Technology, Beijing University of Chemical Technology, Beijing, China; ^3^Department of Radiation Oncology, Huaian No. 2 Hospital, Huaian, Jiangsu Province, China; ^4^Department of Anesthesiology, Shandong Cancer Hospital and Institute, Jinan, Shandong Province, China; ^5^Key Laboratory of Fertility Preservation and Maintenance (Ministry of Education), Ningxia Medical University, Yinchuan, Ningxia, China; ^6^Clinical Laboratory, Shandong Cancer Hospital and Institute, Jinan, Shandong Province, China.

**Supplementary Table 1.** Distribution of selected characteristics among ESCC cases and controls

| Variable | Jiangsu case-control set  (Discovery set) | | |  | Shandong case-control set  (Validation set 1) | | |  |
| --- | --- | --- | --- | --- | --- | --- | --- | --- |
|  | Cases | Controls | *P*-value ^a^ |  | Cases | Controls | *P*-value^a^ |  |
|  | No. (%) | No. (%) |  |  | No. (%) | No. (%) |  |  |
|  | 588 | 600 |  |  | 1000 | 1000 |  |  |
| Sex |  |  | 0.678 |  |  |  | 0.426 |  |
| Male | 413(70.2) | 428(71.3) |  |  | 776(77.6) | 761(76.1) |  |  |
| Female | 175(29.8) | 172(28.7) |  |  | 224(22.4) | 239(23.9) |  |  |
| Age (year)^2^ |  |  | 0.725 |  |  |  | 0.474 |  |
| ≤59(or 56) | 288(49.0) | 300(50.0) |  |  | 516(51.6) | 500(50.0) |  |  |
| >59(or 56) | 300(51.0) | 300(50.0) |  |  | 484(48.4) | 500(50.0) |  |  |
| Smoking status |  |  | <0.001 |  |  |  | <0.001 |  |
| No | 151(25.7) | 397(66.2) |  |  | 248(24.8) | 604(60.4) |  |  |
| Yes | 437(74.3) | 203(33.8) |  |  | 752(75.2) | 396(39.6) |  |  |
| Drinking status |  |  | <0.001 |  |  |  | <0.001 |  |
| No | 254(43.2) | 358 (59.7) |  |  | 447(44.7) | 599(59.9) |  |  |
| Yes | 334(56.8) | 242(40.3) |  |  | 553(55.3) | 401(40.1) |  |  |

Note: ESCC, esophageal squamous cell carcinoma.

^1^Two-sided χ^2^ test.

^2^Median ages of cases for Shandong case-control set and Jiangsu case-control set are 56 and 59 years.

**Supplementary Table 2.** HapMap tag-SNPs in the *MMP13* locus with HCB data of HapMap Rel 27

| **No** | **Test** | **Alleles Captured** |
| --- | --- | --- |
| 1 | rs11225490 | rs11225490, rs10895372, rs3819089 |
| 2 | rs2252070 | rs478927, rs640198, rs2252070 |
| 3 | rs17099788 | rs17099788, rs10502009 |
| 4 | rs3758854 | rs3758854 |

Note: SNP, single nucleotide polymorphism; HCB, Han Chinese Beijing.

**Supplementary Table 3**. Synthetic oligonucleotides used in Electrophoretic Mobility-Shift Assays (EMSA)

| **Name** | **Oligonucleotides (5’→ 3’)** |
| --- | --- |
| Sp1 consensus binding sequence | 5'-ATTCGATCGGGGCGGGGCGAGC-3' |
| *MMP13* rs2252070G | 5'-CCTTCAAGTGACTGGGAAGTGGAAACCT-3' |
| *MMP13* rs2252070A | 5'-CCTTCAAGTGACTAGGAAGTGGAAACCT-3' |

**Supplementary Table 4**. PCR Primers used in construction of luciferase reporter plasmids

| **Plasmid constructs** | **PCR clone Primers (5’→ 3’)** |
| --- | --- |
| pMMP | F: CGGGGTACCTCCCTCAAATTCTACCACAAAC  R: CCGCTCGAGGATTACCTTTACTTTTATAGGC |

Abbreviations: PCR, polymerase chain reaction.
